# A genomic dataset integrating genotyping-by-sequencing, SolCAP array and PCR marker data on tetraploid potato advanced breeding lines

**DOI:** 10.3389/fpls.2024.1384401

**Published:** 2024-05-17

**Authors:** Julien Leuenberger, Sanjeev Kumar Sharma, Karen McLean, Roland Pellé, Aurélie Bérard, Marie-Laure Lesage, Danièle Porhel, Marie-Ange Dantec, Jean-Eric Chauvin, Glenn J. Bryan, Marie-Laure Pilet-Nayel, Marie-Claire Kerlan, Florence Esnault

**Affiliations:** ^1^ Institut de Génétique, Environnement et Protection des Plantes (IGEPP), INRAE, Institut Agro, Univ Rennes, Ploudaniel, France; ^2^ Association des Créateurs de Variétés Nouvelle de Pomme de Terre (ACVNPT), Hanvec, France; ^3^ Cell & Molecular Science Department, The James Hutton Institute, Dundee, United Kingdom; ^4^ Univ Paris Saclay, INRAE, Evry, France

**Keywords:** potato, pre-breeding clones, genomic dataset, genotyping by sequencing, SolCAP DNA array, analysis pipeline

## 1 Introduction

Developing new potato varieties with enhanced resistance to diseases and pests is essential for ensuring food security and promoting sustainable agricultural production. INRAE has been creating potato advanced breeding lines for over 50 years by crossing different wild potato species with *Solanum tuberosum* to improve various traits, such as resistance to major pests and diseases or quality traits. These breeding lines have been distributed to French potato breeders, contributing significantly to the development of more resilient potato varieties. However, further research progress is needed to breed durable multi-resistant potato varieties adapted to low-input (mainly pesticides and fertilizer) agriculture. Molecular markers can effectively assist in pyramiding multiple loci for resistance (Pilet-Nayel et al., 2017) to major diseases and pests of potato, such as late blight disease, cyst nematodes, and viruses, while preserving the agronomic and quality values of varieties.

The development of genomics and the evolution of sequencing methods in the last decade have facilitated the identification of a large number of SNPs (Single Nucleotide polymorphism) from sequencing data. SNPs discovered using EST (Expressed Sequence Tag) or transcriptome data from six potato varieties were used to construct the widely used Infinium 8K SNP SolCAP array (Hamilton et al., 2011; Felcher et al., 2012). However, this genotyping method may display ascertainment bias especially for germplasm containing significant introgressions from wild species genomes. More recently, genotyping-by-sequencing (GBS) approaches were developed, utilizing genome complexity reduction methods. GBS allows the detection of unique SNPs, specific for the variants present within the studied panel, thus offering advantages over the SolCAP array. However, GBS also has some limitations, such as potential biases in genome representation, lower accuracy in detecting rare alleles, and challenges in handling missing data (Negro et al., 2019). Both genotyping approaches are therefore complementary.

Genome-wide association studies (GWAS) have emerged as a powerful tool for identifying genes and genomic regions underlying complex agronomic traits. In potato, Marhadour and Prodhomme (2023) reviewed GWAS studies for the identification of genes and genomic regions involved in resistance to different diseases and pests, or in diverse quality traits. The panel used in this study gathers 285 INRAE tetraploid potato advanced clones originating from different breeding programs mainly focusing on disease and pest resistance. It is therefore a unique set of plant material for performing joint genetic analysis of resistance to multiple diseases and pests and studying its genetic relationships with quality traits. The panel, hereafter, will be referred to as potato MRQ (Multi-Resistance and Quality) panel.

In this study, we developed a potato genomic dataset obtained on the potato MRQ panel using three genotyping techniques: GBS, Infinium 8K SNP SolCAP array and PCR-based markers. This dataset provides a valuable resource for potato researchers and breeders to carry out GWAS analysis on multiple traits, with a particular focus on resistance to multiple diseases and pests.

## 2 Materials and methods

### 2.1 Plant material and DNA extraction

The potato MRQ panel contains 258 advanced breeding clones, 16 commercial varieties and 11 late blight R-gene differentials of Black (originating from *S. demissum*) (Black et al., 1953). The breeding clones were created in various INRAE research programs aimed at (1) enhancing resistance to different pathogens or diseases, including *Phytophthora infestans*, cyst nematodes *Globodera pallida* and *G. rostochiensis*, root knot nematode *Meloidogyne incognita*, *Fusarium* ssp, *Pectobacterium*, Potato Leaf Roll Virus (PLRV), and Potato Virus Y (PVY) and (2) improving quality traits such as cold-induced sweetening or after cooking darkening.

Total genomic DNA was extracted from frozen leaves using a protocol derived from Doyle and Doyle (1990).

### 2.2 Genotyping-by-sequencing

#### 2.2.1 GBS library preparation and sequencing

DNA samples were quantified using the Quant-iTTM PicoGreen^®^ dsDNA Assay Kit (Invitrogen, San Diego, CA). GBS libraries were prepared according to the procedure described by Sharma et al. (2024). Briefly, DNA samples (100 ng each) from the studied panel were processed for double restriction enzyme digestion (*Pst*I-*Mse*I) followed by adapter ligation in a 96-plex (*i.e*., 96 samples per GBS library) multiplexing format. A single *Mse*I adapter was used as common adapter for all samples whereas *Pst*I adapters were barcoded and unique for each sample per library. Processed samples in each GBS library were pooled, PCR-amplified and size-selected (300bp-500bp) using BluePippin™ (Sage Science Inc., Beverly, MA). Quality checks and fragment size analysis on pooled library samples were performed using Bioanalyzer High-Sensitivity DNA chip (Agilent technologies). Each GBS library was sequenced on a single lane of Illumina HiSeq 4k platform to generate 150 bp paired-end sequence reads.

#### 2.2.2 Quality control of raw sequencing data

All FASTQ files were inspected using FastQC to identify any anomalies. The FastQC analysis results revealed no residual sequencing adapters and displayed patterns consistent with libraries generated from DNA subjected to restriction digestion.

#### 2.2.3 Demultiplexing and SNP calling

**Figure 1:**
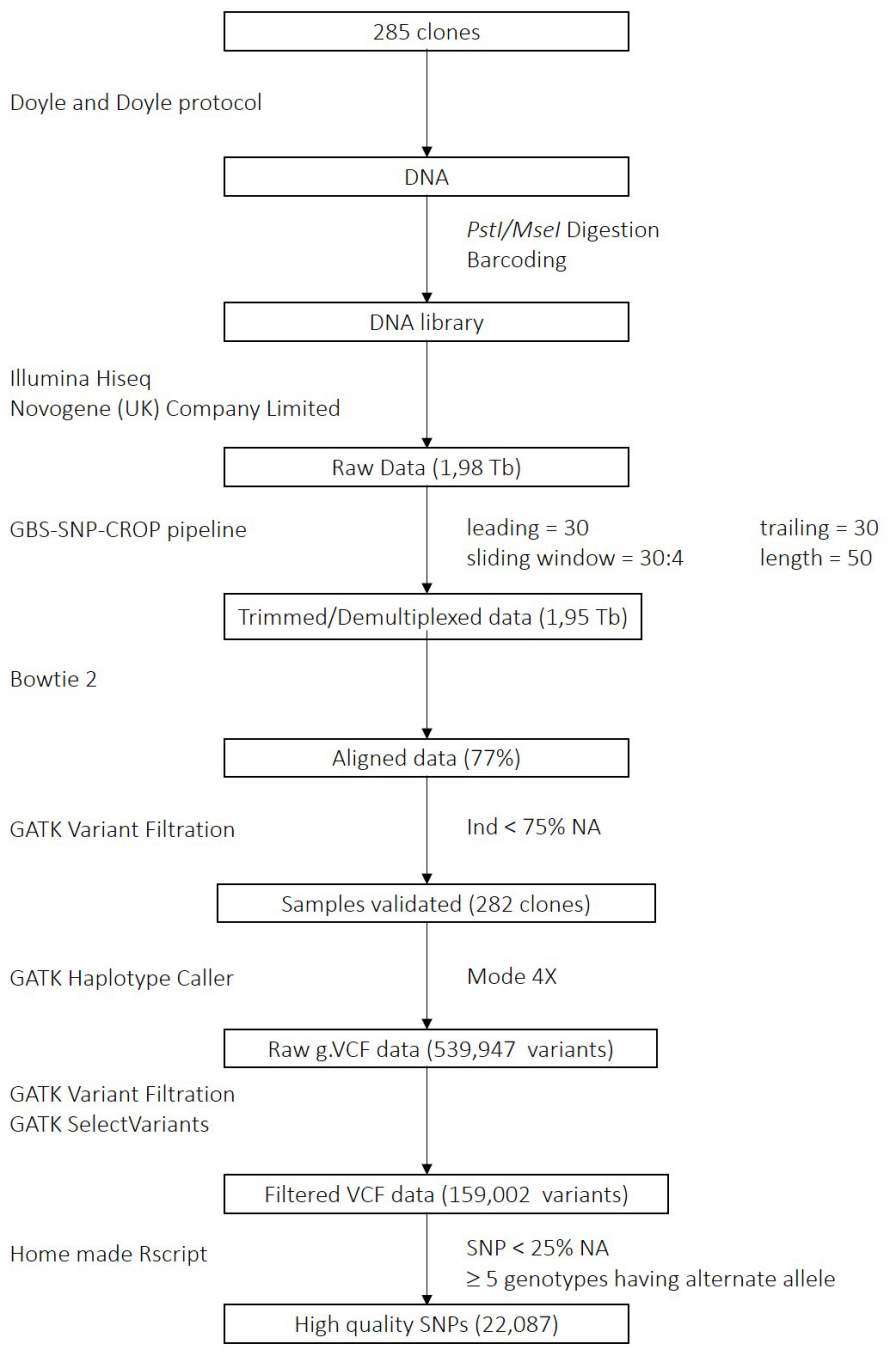
GBS Data Processing Pipeline. Each square represents a processing step; the analysis method and the parameters used are indicated on the left side and on the right side of the scheme, respectively.

We demultiplexed the raw reads and trimmed the adapters using the GBS SNP crop pipeline (Melo et al., 2016) with the following parameters: leading = 30, trailing = 30, sliding window = 30:4, and minimum length = 50. Then, sequences were aligned in paired-end mode to the reference genome *Solanum tuberosum group Phureja* DM 1-3 516 R44 v6.1 using bowtie2 (Langmead and Salzberg, 2012) v2.4.5 with very sensitive mode. Next, we called variants using GATK v4.2.6.1 HaplotypeCaller in mode 4X (Van der Auwera and O’Connor, 2020). Samples with more than 75% of missing data were removed. We then applied filters for biallelic SNPs, QUAL (variant quality) > 30, QD (depth adjusted quality) > 2, GQ (genotype quality) > 20, and DP (coverage depth) > 20. SNPs were then extracted as a matrix with genotypes coded from 0 to 4 based on the dosage of the alternate allele. To further improve the usability of the variants, we filtered for a minimum of 5 genotypes having the alternate allele and removed markers with more than 25% of missing data. The workflow is presented in **Figure 1**.

### 2.3 SolCAP array genotyping and data analysis

A comprehensive quality control (QC) process was conducted for all DNA samples, by examining absorbance and electrophoretic profiles on agarose gels. Additionally, the concentration of each sample was determined using fluorometric measurements with Quant-iT™ PicoGreen^®^ (Invitrogen). Following these assessments, samples were normalized to 50 ng/μl in 96-well plates.

The Infinium 8K SNP array (Felcher et al., 2012) was used following the Infinium HD Assay Ultra Protocol (Illumina, Inc). For each SNP, the assignment of one of the five possible genotypes (AAAA, AAAB, AABB, ABBB, BBBB) to each clone was performed using the FitTetra software (Voorrips et al., 2013) with the Theta value (Carley et al., 2017). To process the genotyping data, default parameters were used (maxn.bin = 200, nbin = 200, sd.threshold = 0.1, p.threshold = 0.99, call.threshold = 0.6, peak.threshold = 0.85). Genotypes were coded as numerical values from 0 to 4 according to the dosage of the alternate allele.

The position of the SNPs on the reference genome DM 1-3 516 R44 v6.1 was attributed according to information available on the Spud DB Potato Genomics Resources website (http://spuddb.uga.edu; DM 1-3 516 R44 - SolCAP 69K and PotVar GBS SNPs - v6.1). Each SNP could be positioned on one of the 12 chromosomes of the reference genome if the sequence had less than 2 mismatches or did not match to multiple positions. The SNP that did not fulfill these conditions were assigned to a fictive chromosome (chromosome 0).

### 2.4 Comparison between GBS and SolCAP array SNP calls

We conducted a thorough evaluation of the consistency and complementarity of SNPs identified using GBS and SolCAP 8K DNA array technologies. The complementarity was checked by evaluating the number of specific variants identified from each technology. To determine the level of concordance between these technologies, we compared, for each common variant, the assignment of one of the five possible genotypes (AAAA, AAAB, AABB, ABBB, BBBB) to each clone, and calculated the percentage of discrepancies.

### 2.5 PCR-based markers

We genotyped the panel using eight dominant PCR-based markers which are commonly used in INRAE marker-assisted selection programs to screen for the presence of gene or QTL for resistance to cyst nematodes *G. pallida* or *G. rostochiensis*. These markers are 57R (Finkers-Tomczak et al., 2011), TG689 (Milczarek et al., 2011; Schultz et al., 2012), ASC151, MS137 (unpublished data), Gpa2-2 (Asano et al, 2012), Gro1-4 (Gebhardt et al., 2006), HC (Sattarzadeh et al., 2006) and Spud1636 (Bryan et al, 2002). The genotyping was performed using protocols adapted from original publications with DNA concentration of about 50 ng/µl. All PCR products were separated by electrophoresis on agarose gel and revealed with ethidium bromide.

We aligned the PCR probe sequences to the reference genome *Solanum tuberosum group Phureja* DM 1-3 516 R44 v6.1 using bowtie2 version 2.4.5, with the very sensitive mode and retained the best alignment position for each marker.

## 3 Results

### 3.1 Genotyping-by-sequencing

The GBS raw data of the potato MRQ panel represented 1.98 terabases. After demultiplexing and trimming, 98% of the reads remained (1.95 terabases). The alignment rate of the reads to the reference genome *Solanum tuberosum* Group Phureja DM 1-3 516 R44 v6.1 was 77%. Three clones showing more than 75% missing data were removed. Variant calling resulted in the identification of 539,947 variants. After applying filters, 159,002 variants remained. Finally, last filters on alternate allele count and missing data produced a dataset consisting in 22,087 high quality SNPs.

### 3.2 SolCAP array genotyping

Out of 2,366,355 total genotyping points, we were able to assign 1,301,718 points. A total of 5053 markers met the criteria set by fitTetra parameters. However, for these markers, some genotypes could not be assigned. Therefore the dataset contains 4.7% of missing data.

From these 5053 SNPs, 764 could not be positioned on any of the 12 potato chromosomes of the reference genome DM 1-3 516 R44 v6.1, leading to their assignment to chromosome 0.

### 3.3 Comparison of GBS and SolCAP array SNP calls

We identified 57 SNPs in common between both methods. Therefore, the majority of these SNPs were unique to each method (4996 SNPs unique to the SolCAP DNA array and 22087 SNPs unique to GBS). Upon closer inspection of these common positions, we observed that 34 SNPs displayed the same polymorphism, while 23 displayed complementary patterns (e.g., in GBS [A/C], in SolCAP [T/G]).

**Figure 2:**
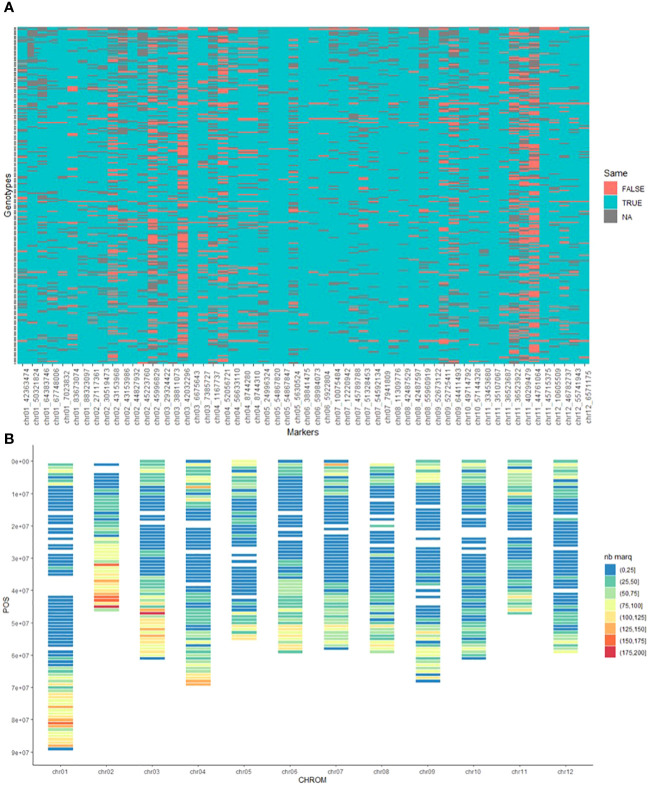
**(A)** Heatmap Depicting Concordance and Discordance in genotype calls of the SNPs in common between GBS and SolCAP array. Red (False): discordance, Blue (True): concordance, Grey (NA): missing data, **(B)** Distribution of the 26,384 identified SNPs along the chromosomes of Potato. Color scale from blue representing a density of 0 to 25 markers per 100 kb to red representing a density of 175 to 200 markers per 100 kb.

The overall concordance rate between the two genotyping methods (12289 comparison points) was found to be 90% (**Figure 2A**), which is consistent with the findings of Bastien et al. (2018). The observed discordance varied significantly among the markers, with some showing no discordance at all (e.g., chr11_35107067), while others displayed a high number of discordant genotypes, such as chr11_44761064 with 111 discordant genotypes (53%). Furthermore, we identified 6 markers that displayed each more than 30% discordance, representing 44% of the total discordances. Upon deeper investigation, we found that 80% of the discordant genotype calls occurred when the alternate allele was not detected using the GBS method. These discrepancies could be attributed to technical factors such as insufficient sequencing depth or biological factors like the presence of a variant within the restriction site. The remaining 20% discordant genotype calls were due to different allelic dosage of heterozygous genotypes.

### 3.4 Final matrix construction

All the genotyping data were merged to obtain the final matrix, resulting in a total of 27,148 markers (22,087 SNPs from GBS, 5,053 SolCAP SNPs, and 8 PCR based markers). When markers were identified in common by GBS and SolCAP array, both markers were kept in the final matrix.

## 4 Discussion

The average marker density on the genome, whose size is estimated at 844 Mb (Pham et al., 2020), was one SNP every 31kb. However, the markers were not evenly distributed along the whole genome (**Figure 2B**). None or few markers were identified in the centromeric regions, which can be explained by the use of the restriction enzyme *Pst*I which is sensitive to methylation. The genomic distribution of the different types of markers was also examined and no visible patterns were detected.

To verify the consistency of the genomic dataset, we conducted a linkage disequilibrium (LD) analysis using the methods described by Vos et al. (2017). The LD values ranged from 0.1 to 1.4 Mb for LD_1/2 90_ and from 2.0 to 11.7 Mb for LD_1/10 90_, depending on the chromosome, which was consistent with those reported by Sharma et al. (2018). The average interval between two SNPs being lower than the estimated LD values, our dataset guarantees extensive genome coverage for the discovery of marker-trait associations across the genome and the building of haplotype blocks at targeted loci.

The extensive SNP distribution of this dataset will enable breeders to perform GWAS for a diversity of traits as the ones mentioned in Marhadour & Prodhomme (2023), especially those relating to disease and pests resistance and quality. It will also support marker-assisted selection efforts (Islam et al., 2024). Breeders could also use this dataset to perform genomic predictions of breeding values for traits of interest (Gemenet et al., 2020) and identify good genitors among the genotypes of the potato MRQ panel for crossings in genomic selection schemes (Pandey et al., 2023).

The scientific community will also benefit from this dataset as a great tool for identifying ancestral haploblocks (Wang et al., 2022), which are notably difficult to discern in autotetraploid and highly heterozygous species like potato. The high number and diversity of genetic markers in this panel provide a robust groundwork for the creation and refinement of computational strategies and techniques for precise phasing in tetraploid potatoes.

## 5 Conclusion

The data reported in this study represents a comprehensive genomic dataset for 282 tetraploid potato clones, predominantly comprising INRAE advanced breeding lines for resistance to multiple pests and diseases, by integrating genotyping data derived from GBS, SolCAP 8k DNA array, and PCR-based markers. The final dataset includes 27,148 high-quality SNPs, providing a valuable resource for potato researchers and breeders especially working on resistance to biotic stresses.

## Data availability statement

The data presented in the study are deposited in the Omics Dataverse repository accession number doi: 10.57745/DPHPOR.

## Author contributions

JL: Conceptualization, Data curation, Formal analysis, Investigation, Methodology, Validation, Visualization, Writing – original draft. SS: Methodology, Supervision, Writing – review & editing. KM: Investigation, Writing – review & editing. RP: Resources, Writing – review & editing. AB: Investigation, Writing – review & editing. ML: Investigation, Writing – review & editing. DP: Investigation, Writing – review & editing. MD: Resources, Writing – review & editing. JC: Funding acquisition, Writing – review & editing. GB: Supervision, Writing – review & editing. MP: Supervision, Writing – review & editing, Project administration, Conceptualization, Funding acquisition. MK: Supervision, Writing – review & editing, Project administration, Conceptualization, Funding acquisition, Resources. FE: Funding acquisition, Project administration, Supervision, Writing – review & editing, Conceptualization, Resources.
